# Cryoballoon Ablation With the POLARx FIT or the Arctic Front Advance Pro for Paroxysmal Atrial Fibrillation: A Health Economic Analysis

**DOI:** 10.36469/001c.133223

**Published:** 2025-04-21

**Authors:** Luigi Pannone, Steffen Uffenorde, Antonia Bosworth Smith, Domenico Giovanni Della Rocca, Pasquale Vergara, Ioannis Doundoulakis, Antonio Sorgente, Alvise Del Monte, Giacomo Talevi, Ingrid Overeinder, Gezim Bala, Alexandre Almorad, Erwin Ströker, Juan Sieira, Ali Gharaviri, Mark La Meir, Pedro Brugada, Andrea Sarkozy, Gian Battista Chierchia, Carlo de Asmundis

**Affiliations:** 1 Heart Rhythm Management Centre, Postgraduate Program in Cardiac Electrophysiology and Pacing, Universitair Ziekenhuis Brussel; Vrije Universiteit Brussel, European Reference Networks Guard-Heart, Brussels, Belgium; 2 Boston Scientific Medizintechnik GmbH, Düsseldorf, Germany; 3 Coreva Scientific GmbH & Co KG, Koenigswinter, Germany; 4 Cardiac Surgery Department, Universitair Ziekenhuis Brussel; Vrije Universiteit Brussel, European Reference Networks Guard-Heart, Brussels, Belgium

**Keywords:** atrial fibrillation, pharmacoeconomy, cryoballoon catheter, catheter ablation, pulmonary vein isolation

## Abstract

**Background:** Pulmonary vein isolation (PVI) is the main ablation strategy for the treatment of paroxysmal atrial fibrillation. Different technologies are available for PVI, including various cryoballoon catheters (CB-A). Compared with the Arctic Front Advance Pro™, the novel POLARx FIT™ CB-A might reduce costs for atrial fibrillation ablation. **Objective:** The aim of this study is to perform a health economic evaluation of two cryoballoon systems for PVI procedures. **Methods:** All patients undergoing their first PVI procedure with POLARx FIT™ CB-A or the Arctic Front Advance Pro™ CB-A were prospectively enrolled. The health economic analysis was performed on the index hospitalization and procedure. The primary safety endpoint included procedure-related adverse events within the index hospitalization. A decision tree model was built to estimate downstream costs. **Results:** A total of 80 patients with paroxysmal atrial fibrillation undergoing PVI were analyzed, with 40 patients in each arm. Compared with the Arctic Front Advance Pro™ CB-A, POLARx FIT™ CB-A showed a lower procedure time, left-atrium dwell time, and fluoroscopy time. The complication rate was low (6.3%) and included 3 reversible phrenic nerve palsies in the POLARx FIT™ CB-A group vs 2 in the Arctic Front Advance Pro™ CB-A group. Compared with the Arctic Front Advance Pro, the POLARx FIT™ CB-A was associated with lower procedural costs (€2069.7 ± €165.2 vs €2239.5 ±  €366.0; *P* =.009). **Conclusion:** The POLARx FIT™ CB-A was associated with a shorter procedure time, translating into lower procedural costs, compared with the Arctic Front Advance Pro. Complications were rare and comparable between the two technologies.

## INTRODUCTION

Atrial fibrillation (AF) is the most common sustained cardiac arrhythmia.^1^ It has a significant cost burden; patients with AF have an estimated mean total healthcare cost (per patient per year) approximately $27 896 more than the non-AF cohort.^2^

Different strategies exist for AF management, including rate and rhythm control. Among rhythm control, catheter ablation has been demonstrated to be the most cost-effective therapeutic choice, especially in high-income countries^3^ and in patients with heart failure.^4,5^

Pulmonary vein isolation (PVI) is currently recommended as the primary lesion set for all patients undergoing AF ablation.^6^ PVI can be performed with radiofrequency, cryoballoon catheter (CB-A) or, recently, with pulsed field ablation. Different clinical trials demonstrated no difference in clinical outcomes between the three technologies.^7,8^

CB-A has been shown as a highly cost-effective strategy for AF compared with first-line anti-arrhythmic drug treatment in different countries, including the United Kingdom^9,10^ and Germany.^11^

Different CB-A technologies are available, including the Arctic Front Advance Pro™ CB-A system (Medtronic Inc.) and, more recently, the POLARx™ CB-A and the POLARx FIT™ CB-A (Boston Scientific). The latter is a novel CB-A with a balloon diameter that can be expanded from 28 to 31 mm.^12^

The POLARx™ CB-A system has been associated with a shorter procedural time than the Arctic Front Advance Pro™ CB-A. This might translate to lower procedural cost and higher cost-effectiveness for the POLARx™ system. However, there are currently no comparative data between the novel POLARx FIT™ and the Arctic Front Advance Pro™ systems. Furthermore, no pharmacoeconomic analysis has been performed between these two CB-A systems.

Thus, the aims of this study are (1) to compare the novel POLARx FIT™ and the Arctic Front Advance Pro™ systems in terms of procedural time and safety and (2) to perform a health economic analysis to evaluate the procedural costs of the two systems.

## METHODS

### Study Population

All consecutive patients undergoing an index ablation for paroxysmal AF at Universitair Ziekenhuis Brussel, Belgium, between 2023 and 2024, were prospectively screened and enrolled if they met the following inclusion criteria:

- Paroxysmal AF diagnosis following current guidelines^1^
- First AF ablation procedure with PVI by means of CB-A

The same experienced operators performed the procedures in both groups. All patients underwent PVI with the POLARx FIT™ (Boston Scientific) or the Arctic Front Advance Pro™ CB-A system (Medtronic Inc.). The exclusion criteria were as follows:

- Previous left-side ablation
- Ablation lesion set other than PVI, except for cavotricuspid isthmus (CTI) ablation
- Use of different ablation technologies, except for radiofrequency to perform CTI line
- Intracavitary thrombus

The following data were collected from the electronic medical record:

- Demographic data
- Comorbidities data
- Echocardiography and anti-arrhythmic drug data
- Procedural resources used

In particular, the following data were collected to posteriorly calculate procedural costs: (1) catheterization laboratory resources per hour (ie, physicians, nurses, anesthesia), (2) hospitalization (ie, length of hospital stay in the general ward and ICU), and (3) acute complications. Data were carefully reviewed and confirmed by 2 independent researchers (L.P. and I.D.), both blinded to cardiac arrhythmia recurrence, to guarantee the accuracy of the data extraction.

The study complied with the Declaration of Helsinki as revised in 2013; the ethics committee approved the study. All patients signed an informed consent that had been approved by the institutional review board.

### Ablation Procedure With Cryoballoon Catheter

All patients underwent PVI with POLARx FIT™ or the Arctic Front Advance Pro™ CB-A system. The standard preprocedural management and ablation for CB-A has been previously described in detail.^13^

All procedures were performed on patients under general anesthesia with esophageal temperature monitoring. Cryo-energy applications were interrupted if luminal esophageal temperatures dropped below 15° C. After obtaining access to the left atrium through a steerable sheath (POLARSHEATH™ [Boston Scientific] 15.5F or FlexCath Advance [Medtronic Inc.] 15F), a 28 mm CB-A catheter (POLARx FIT™ Balloon Catheter or Arctic Front Advance Pro™ ) was advanced in the left atrium for PVI, and an inner lumen mapping catheter (POLARMap [Boston Scientific] or Achieve [Medtronic Inc.]) was positioned in each pulmonary vein ostium. The CB-A was then advanced, inflated, and positioned at the pulmonary vein ostia. Optimal vessel occlusion was confirmed by selective contrast injection. Once satisfactory vessel occlusion was achieved, cryo-energy delivery was initiated. Standard cryothermal applications lasted 180 seconds, targeting a temperature of -40° C within the first 60 seconds. If the temperature target was not achieved, the cryothermal application was prolonged to 240 seconds or an additional cryoapplication was performed. To prevent phrenic nerve palsy, diaphragmatic stimulation was achieved by pacing the phrenic nerve during right PV ablation.

During the entire procedure, activated clotting time was maintained over 300 seconds by supplementing heparin infusion as required.

### Postprocedural Management and Endpoints

All patients underwent continuous telemetry monitoring for at least 24 hours after the procedure and were discharged after overnight observation if no complications occurred. Before discharge, all patients received transthoracic echocardiography and venous Doppler ultrasound. Oral anticoagulation was initiated on the evening of the ablation and continued for at least 2 months post procedure, then adjusted based on the patient’s thromboembolic risk profile. Anti-arrhythmic drugs were continued for 2 months after ablation and thereafter based on clinical judgment and patient preference.

The primary safety endpoint was defined as any major periprocedural complication (eg, death, stroke, pericardial effusion/tamponade requiring treatment, ischemic events, diaphragmatic paralysis, pericarditis, and vascular complications requiring treatment).

The primary endpoint was procedural costs (excluding materials), including catheterization laboratory resources per hour (ie, physicians, nurses, anesthesia), hospitalization costs (ie, length of hospital stay in the general ward and intensive care unit, exams), and acute complications.

### Health Economic Model

**Figure 1.**
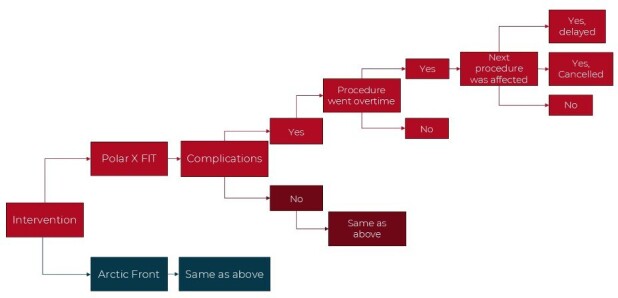
Pharmacoeconomic Decision Tree Model

A decision tree model was built in Microsoft Excel to compare the two CB-A systems, including delay and cancellation costs over a 1-year time horizon (**Figure 1**). In the model, costs and resource use were captured for a hypothetical cohort of 105 patients (informed by the rate of the procedures during this data collection). The hospital perspective was used, as this was a single-center hospital study. Economic outcomes per procedure were modeled and the primary outcome was the total cost, which included the cost of delays and staffing time costs.

Delay (or overtime) was defined as observed procedure time higher than expected procedure time. Delay time was defined as the difference between expected and observed procedure time. Cancellation due to delay was defined as a procedure not performed due to delay of previous procedure. Cancellation cost due to delay was set at €1962.^14^ As no Belgian data were reported in this study, the median value of the range was used as an approximation. Due to a lack of available data, the cost of a delay was assumed to be 20% of a cancellation. To account for the uncertainty in the delay cost, the input was varied from 10% to 50% of a cancellation. All costs were inflated to 2023 costs. The details of the input parameters are shown in **Table 1**. Input data were sourced from this data collection center when possible and supplemented with unit costs from the literature. As the model had a 1-year time horizon, no discount rate was used for the costs. When the variance was unknown an assumed variance of 20% was used. A probabilistic sensitivity analysis (PSA) was performed with 1000 Monte Carlo simulations. The results of the PSA informed the 95% credible intervals (CrIs). A 1-way sensitivity analysis was performed while varying the inputs by 10%, to determine which had the largest impact on the model.

**Table 1.** Model Inputs

**Input Parameter**	**Value**	**Unit**	**Low**	**High**
General				
Population	105		0.00	0.00
POLARx FIT™ group				
Average procedure time	63	Minimum	56.30	70.00
Procedures that go overtime	0	Percentage	0.00	0.03
Average overtime procedure time	0	Minimum	0.00	0.00
Next procedure delay	0.00	Percentage	0.00	0.03
Next procedure cancelled	0	Percentage	0.00	0.03
Doctor workload factor	2.8		2.21	3.32
No. of doctors	3		2.40	3.20
No. of nurses	1		0.00	0.00
No. of anesthesiologists	1		0.00	0.00
Arctic Front Advance™ group				
Average procedure time	69	Minimum	60.00	88.80
Average overtime procedure time	120	Minimum	0.00	0.00
Procedures that go overtime	6.0	Percentage	0.00	0.00
Next procedure delay	33.0	Percentage	0.00	0.00
Next procedure cancelled	0.0	Percentage	0.00	0.03
Doctor workload factor	2.7		2.04	3.42
No. of doctors	3		2.20	3.20
No. of nurses	1		0.00	0.00
No. of anesthesiologists	1		0.00	0.00
Costs^a^				
Cost of cancellation^14^	1962	€	217	3168
Delay cost as a proportion of the cancellation cost	20	Percentage	10.00	50.00
Cost of physician	119	€	0.00	0.00
Cost of anesthesiologist	119	€	0.00	0.00
Cost of nurse	57	€	0.00	0.00

^a^Cost inputs were inflated to 2023 euros.

### Statistical Analysis

All variables were tested for normality with the Shapiro-Wilk test. Normally distributed variables were described as mean ± SD, and the groups were compared through analysis of variance (ANOVA) and paired or unpaired *t*-test as appropriate, while the non-normally distributed variables were described as median (interquartile range) and compared by Mann-Whitney test as appropriate. The categorical variables were described as frequencies (percentages) and compared by Fisher’s exact test as appropriate. A *P* value less than .05 was considered statistically significant. The analysis was performed using R software, version 3.6.2.

## RESULTS

### Study Population Characteristics

Eighty consecutive patients were included and analyzed in the study: 40 patients in the POLARx FIT™ group and 40 patients in the Arctic Front Advance Pro™ group. Complete patient characteristics showed no differences in demographic characteristics, comorbidities, or echocardiography data between the two groups (**Table 2)**. In particular, CHA2DS2-VASc score was similar between the POLARx FIT™ group (1.9 ± 1.3) and the Arctic Front Advance Pro™ group (1.8 ± 1.4) (*P* = .62). At echocardiographic evaluation, left-atrium volume index (LAVi) was 43.4 mL/m^2^ ± 6.7 for the POLARx FIT™ group vs 42.5 mL/m^2^ ± 12.8 for the Arctic Front Advance Pro™ group (*P* = .84).

**Table 2.** Clinical Characteristics of Patients

	**POLARx FIT™ Group (n = 40)**	**Arctic Front Advance™ Group (n = 40)**	**Total (N = 80)**	***P* Value**
Age (y) at ablation (n, %)				.79
30-39	2 (2.5)	0 (0.0)	1 (1.2)	
40-49	4 (10.0)	2 (5.0)	6 (7.5)	
50-59	7 (17.5)	10 (25.0)	17 (21.2)	
60-69	16 (40.0)	19 (47.5)	35 (43.8)	
70-79	9 (22.5)	7 (17.5)	16 (20.0)	
80-89	3 (7.5)	2 (5.0)	5 (6.2)	
Male, n (%)	23 (57.5)	23 (57.5)	46 (57.5)	1.00
AF paroxysmal, n (%)	40 (100)	40 (100)	80 (100)	1.00
CHA2DS2-VASc score	1.9 ± 1.3	1.8 ± 1.4	1.9 ± 1.4	.62
LVEF, %	54.9 ± 9.0	55.1 ± 8.6	55.0 ± 8.7	.93
LAVi (mL/m^2^)	43.4 ± 6.7	42.5 ± 12.8	42.9 ± 10.3	.84
TIA or stroke, n (%)	5 (12.5)	3 (7.5)	8 (10.0)	.71
CAD, n (%)	6 (15.0)	4 (10.0)	10 (12.5)	.74
AADs before ablation	16 (40.0)	14 (35.0)	30 (37.5)	.82
DOAC, n (%)	40 (100.0)	40 (100.0)	80 (100.0)	1.00

Abbreviations: AADs, anti-arrhythmic drugs; AF, atrial fibrillation; CAD, coronary artery disease; DOAC, direct oral anticoagulants; LAVi, left-atrium volume index; LVEF, left ventricular ejection fraction; TIA, transient ischemic attack

There was no significant difference in anti-arrhythmic drug treatment between the two groups (16 patients [40.0%] vs 14 patients [35.0%] [*P* = .82], respectively) (**Table 2**).

### Procedural Characteristics

**Figure 2.**
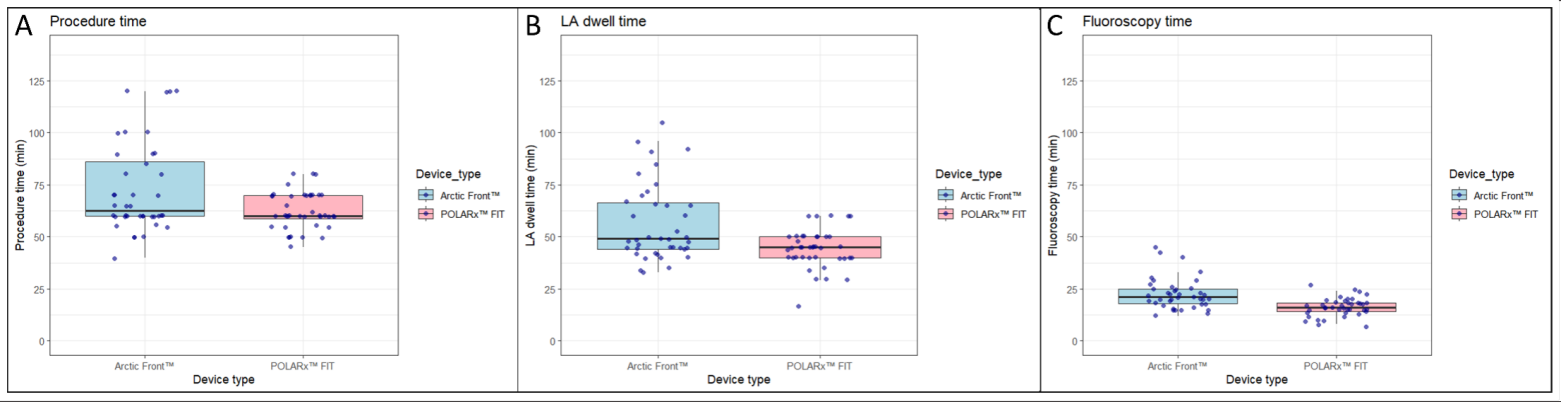
Box Plots for Procedural Characteristics: POLARx FIT™ Group vs Arctic Front Advance Pro™ Group Abbreviation: LA, left atrium.

PVI was performed with success in all 80 (100%) patients. Compared with the Arctic Front Advance PRO, the POLARx FIT™ was associated with a shorter skin-to-skin procedure time (62.7 ± 8.9 minutes vs 72.7 ± 21.6 minutes; *P* = .008), a shorter left-atrium dwell time (44.3 ± 9.2 minutes vs 56.3 ± 18.5 minutes; *P* <.001) and a shorter fluoroscopy time (16.3 ± 4.4 minutes vs 22.5 ± 7.5 minutes; *P* < .001) (**Figure 2** and **Table 3**).

Compared with the Arctic Front Advance Pro™, the POLARx FIT™ was associated with (**A**) a shorter skin-to-skin procedure time (62.7 ± 8.9 min vs 72.7 ± 21.6 min, *P* = .008), (**B**) a shorter left-atrium dwell time (44.3 ± 9.2 min vs 56.3 ± 18.5 min, *P* < .001), and (**C**) a shorter fluoroscopy time (16.3 ± 4.4 min vs 22.5 ± 7.5 min, *P* < .001).

**Table 3.** Procedural Characteristics

	**POLARx FIT™ Group (n = 40)**	**Arctic Front Advance™ Group (n = 40)**	**Total (N = 80)**	***P* Value**
Skin-to-skin procedure time (min)	62.7 ± 8.9	72.7 ± 21.6	67.7 ± 17.2	.008
LA dwell time (min)	44.3 ± 9.2	56.3 ± 18.5	50.3 ± 15.7	< .001
Fluoroscopy time (min)	16.3 ± 4.4	22.5 ± 7.5	19.4 ± 6.8	< .001
CTI performed, n (%)	4 (10.0)	4 (10.0)	8 (10.0)	1.00
Decrease in phrenic nerve, n (%)	3 (7.5)	2 (5.0)	5 (6.2)	1.00
Phrenic nerve palsy, n (%)	0 (0.0)	0 (0.0)	0 (0.0)	1.00
Primary safety outcome, n (%)	0 (0.0)	0 (0.0)	0 (0.0)	1.00

Abbreviations: CT, cavotricuspid isthmus ablation; LA, left atrium.

All patients were in sinus rhythm at the beginning and at the end of the procedure. A transient decrease in phrenic nerve capture occurred in 3 patients (7.5%) in the POLARx FIT™ group vs 2 patients (5.0%) in the Arctic Front Advance Pro™ group (*P* = 1.00), with complete resolution before the end of the procedure. No deaths, pericardial effusion/tamponade requiring treatment, ischemic events, cerebrovascular events, or vascular complications requiring treatment occurred (**Table 3**).

### Health Economic Analysis

Compared with the Arctic Front Advance Pro™ CB-A, POLARx FIT™ CB-A was associated with lower procedural costs (€2069.7 ± €165.2 vs 2239.5 ± €366.0; *P* = .009). This corresponds to a decrease in overall costs of -7.5% and was driven by both lower procedural time and lower procedural delay/cancellation in the POLARx FIT™ CB-A group. In particular, the mean procedural delay (overtime) was 7.2 minutes in the Arctic Front Advance Pro™ CB-A vs 0.0 minutes in the POLARx FIT™ CB-A group (*P* < .001).

**Figure 3.**
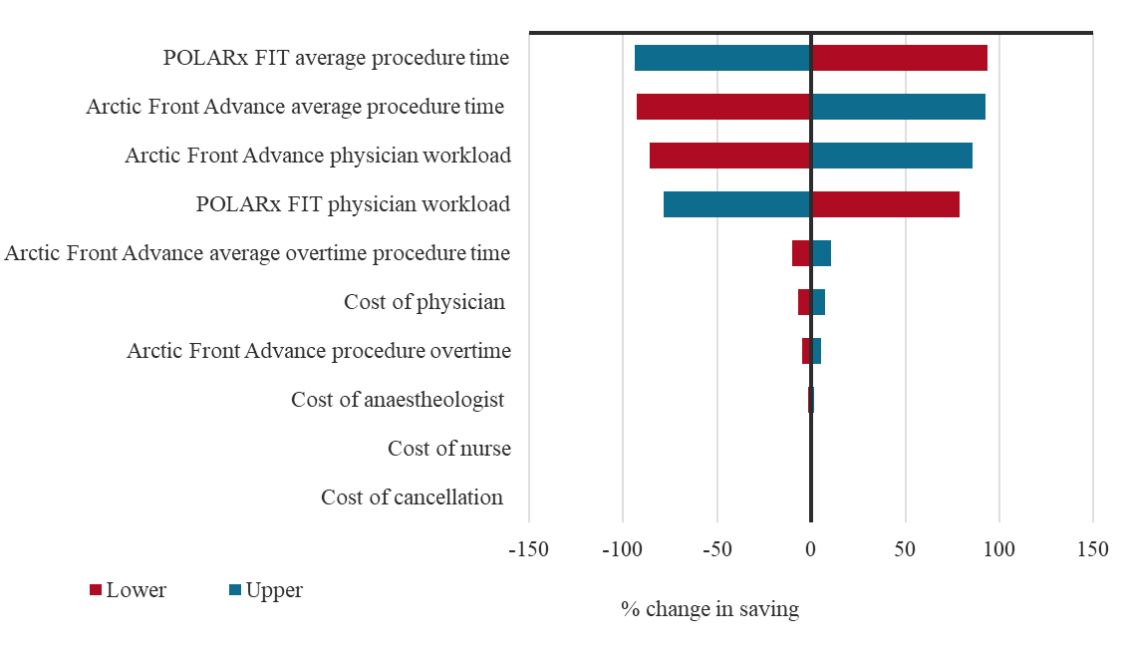
Tornado Plot of 1-Way Sensitivity Analysis

The PSA analysis of the economic model reported that the median cost difference between the two groups was €123.40 (95% CrI: €30.80-€393.90), with 99.9% of simulations yielding a positive difference in favor of the POLARx FIT™ CB-A (**Table S1**). One-way sensitivity analysis results are presented in a tornado plot, which illustrates the percentage change in the total costs when varying the input by 10% (**Figure 3**). Procedure time and physician workload were the main drivers in the model.

## DISCUSSION

The main findings of this study can be summarized as follows:

- Compared with the Arctic Front Advance Pro™ CB-A, the POLARx FIT™ CB-A was associated with a shorter skin-to-skin procedure time, a shorter left-atrium dwell time, and a shorter fluoroscopy time.
- Compared with the Arctic Front Advance Pro™ CB-A, the POLARx FIT™ CB-A was associated with lower procedural costs (€2069.7 ± €165.2 vs €2239.5 ± €366.0; *P* = .009), corresponding to a 7.5% decrease in overall costs.

The results of the 1-way sensitivity analysis illustrate the importance of reducing procedure time, as this can be a significant driver of costs in ablation procedures.

### Health Economic Impact of Cryoballoon Catheter Ablation

CB-A has been shown as a highly cost-effective strategy for AF management compared with first-line anti-arrhythmic drug treatment. In a German study, results from the economic analysis estimated CB-A to be associated with an increase in cost of €191 per patient over a lifetime compared with anti-arrhythmic drugs, while offering an increase in quality of life.^11^ In particular, CB-A had a 98.0% probability of being cost-effective. This has been replicated in UK studies showing a 45% relative reduction in AF burden, translating into higher cost-effectiveness of CB-A compared with anti-arrhythmic drugs.^9^

Although CB-A is a cost-effective strategy, CB-A workflow and technology play a major role in its procedural pharmacoeconomic impact. Indeed, procedural time is a major factor determining procedural costs.^15^ In the AVATAR trial evaluating an optimized CB-A protocol, the within-trial cost analysis showed that the optimized AVATAR protocol was cost-saving (£1279/patient) compared with the conventional ablation protocol.^16^ A recent study compared the procedural costs (excluding materials) of the 3 major energy sources for AF catheter ablation: pulsed field ablation, radiofrequency and CB-A.^17^ The index procedure mean cost was €2222.2 for CB-A, €2659 for radiofrequency, and €1741.1 for pulsed field ablation. These findings were driven by a reduction in the procedural time with CB-A and with pulsed field ablation.

The current study is in line with previous results,^17^ showing a procedural cost of €2069.7 for the POLARx FIT™ CB-A, less than €2239.5 for the Arctic Front Advance Pro™ CB-A. Thus, the POLARx FIT™ CB-A system demonstrated a favorable cost profile, lying between CB-A and pulsed field ablation. This was secondary to a significant reduction in the procedural time for the POLARx FIT™ CB-A group. The current study is the first to compare acute procedural outcomes and health economic outcomes between these two systems. Our data are consistent with previous research from our group showing a shorter skin-to-skin procedure time, a shorter left-atrium dwell time, and a shorter fluoroscopy time for the POLARx CB-A compared with the Arctic Front Advance Pro™ CB-A, with a similar safety profile.^18^

The study design focuses on short-term outcomes. However, a recent randomized clinical trial comparing the POLARx and the Arctic Front CB-A for AF ablation found no difference between the two in terms of AF recurrence, repeat ablation, and medication use at 1-year follow-up.^19^

Future studies should assess the cost savings associated with using interventional methods such as cryoballoon ablation compared with noninterventional rhythm care management.

### Limitations

The current study is a single-center study from a high-volume center experienced in AF ablation, and findings may not be generalizable to other settings. Therefore, variations in hospital unit costs and patient population treated might affect the results in other centers. Moreover, the physician’s experience with using the different devices might also affect the overall duration of the procedure, as a learning curve is expected with using new technologies. The sample size is relatively small. The same experienced operators performed the procedures in both groups. The main limitation is lack of randomization. However, there was no difference in baseline characteristics between the two groups, including atrial dimensions. This study has a hospital perspective, and as such does not capture all related costs such as pre- and post-procedural care and follow-up and management of complications. The cost of the ablation material was not included in the analysis, as these are limited by the hospital-specific purchasing contracts. This can have a substantial impact on the cost of the procedure and the overall cost-effectiveness of the interventions. Our results showed that POLARx FIT™ CB-A may be considered cost-saving compared with Arctic Front Advance Pro™ CB-A if the difference between the unit cost of the devices does not exceed €393.90. This result assumes that a hospital would perform the same number of procedures per day regardless of the device used. The reduced procedure time and variability for POLARx FIT™ CB-A shown in this analysis may indicate that the hospital can complete more procedures per day with this device. This would increase hospital efficiencies, results in more hospital revenue, and potentially reduce unnecessary shifts in OR schedules. When used in this way, POLARx FIT™ CB-A may remain cost-saving at a higher differential cost. The present study collected only short-term outcomes, without collecting atrial fibrillation recurrence and redo rates. The focus here was on single hospital costs, and long-term outcomes may not result in patients returning to the same hospital. These long-term outcomes should be collected when considering a health service perspective.

## CONCLUSIONS

In patients with AF undergoing an index catheter ablation, compared with the Arctic Front Advance Pro™ CB-A, the use of the POLARx FIT™ CB-A is associated with a shorter skin-to-skin procedure time, a shorter left-atrium dwell time, and a shorter fluoroscopy time. This translates into higher cost-effectiveness for the POLARx FIT™ CB-A system with a cost reduction of approximately 7% to 8%.

### Disclosures

A.B.S. is an employee of Coreva Scientific GmbH & Co. KG, which received consultancy fees from Boston Scientific. S.U. is an employee of Boston Scientific, which is the manufacturer of the POLARx FIT™ system. A﻿.﻿So. received research grants from Daiichi-Sankyo and Bayer; he has received speaker fees from Menarini and Bayer. M.L.M. is a consultant for Atricure. A.S. is a consultant for Biosense Webster and Medtronic and received speaker fees from Biosense Webster, Biotronik, Pfizer, and Microport. G.C. received compensation for teaching purposes and proctoring from Medtronic, Abbott, Biotronik and Boston Scientific. C.d.A. receives research grants on behalf of the center from Biotronik, Medtronic, Abbott, Microport, Boston Scientific, and AtriCure; C.d.A. received compensation for teaching purposes and proctoring from Medtronic, Abbott, Biotronik, Microport, Boston Scientific, Atricure and Daiichi Sankyo. The Universitair Ziekenhuis Brussel received fees for data collection.

### Data Availability

The data underlying this article will be shared by the corresponding author upon reasonable request.

## Supplementary Material

Online Supplementary Material
