# Supplementary material for: Cryoballoon Ablation With the POLARx FIT or the Arctic Front Advance Pro for Paroxysmal Atrial Fibrillation: A Health Economic Analysis
Source: J Health Econ Outcomes Res. 2025 Apr 21;12(1):155–61. doi: 10.36469/001c.133223 (PMC12017263; doi:10.36469/001c.133223)
Supplement: Online Supplementary Material [file jheor_2025_12_1_133223_279654.pdf]

Journal of Health Economics and Outcomes Research

Online Supplementary Material

Cryoballoon Ablation With the POLARx FIT™ or the Arctic Front Advance Pro™ for Paroxysmal Atrial Fibrillation: A Health Economic Analysis. *JHEOR*. 2025;12(1):155-163. doi:10.36469/jheor.2025.133233

Table S1: Phamacoeconomic Model: Probabilistic Sensitivity Analysis Results

This supplementary material has been provided by the authors to give readers additional information about their work.

This is an open-access article distributed under the terms of the Creative Commons Attribution 4.0 International License (CCBY-4.0). View this license's legal deed at http://creativecommons.org/licenses/by/4.0 and legal code at http://creativecommons.org/licenses/by/4.0/legalcode for more information.

Table S1. Phamacoeconomic Model: Probabilistic Sensitivity Analysis Results

PSA Results	Median Difference (Crl 95%)	Simulations in the Model With Positive Difference (%)
Total time (min)	9.3 (5.2-21.4)	100
Overtime (min)	7.1 (4.1-11.3)	100
Not overtime (min)	2.0 (-0.8-13.9)	87.9
Staffing time cost (€)	114.90 (22.30-382.90)	99.7
Cost of delays (€)	7.30 (3.70-14.60)	100
Total cost (€)	123.40 (30.80-393.90)	99.9

Abbreviations: Crl, credible interval; PSA, probabilistic sensitivity analysis.
